# Diesel exhaust in miners study: how to understand the findings?

**DOI:** 10.1186/1745-6673-7-10

**Published:** 2012-06-07

**Authors:** Peter Morfeld

**Affiliations:** 1 Institute for Occupational Epidemiology and Risk Assessment of Evonik Industries, Essen, Germany; 2 Institute and Policlinic for Occupational Medicine, Environmental Medicine and Prevention Research, University of Cologne, Cologne, Germany

**Keywords:** Diesel exhaust in miners study, Epidemiology, Lung cancer, Respirable elemental carbon

## Abstract

The Diesel Exhaust in Miners Study (DEMS) is an outstanding epidemiological project on the association between occupational diesel exhaust exposures, measured as long-term respirable elemental carbon (REC) estimates, and lung cancer mortality in a large cohort of US miners. Two articles published recently (Attfield et al. (J Natl Cancer Inst Epub, [2012]), Silverman et al. (J Natl Cancer Inst Epub, [2012])) dsescribed the epidemiological findings. These papers are expected to have considerable impact on the evaluation of the carcinogenic potential of diesel exhaust and, furthermore, on occupational and environmental limit value discussions related to diesel motor emissions and particle exposures. DEMS found remarkable exposure-response relationships between REC exposure estimates and lung cancer mortality - conditional on a pronounced effect of surface vs. underground work on lung cancer risk. If this risk factor is ignored the estimated REC-lung cancer association is attenuated substantially. The authors relied on this risk factor in their main analyses. However, this factor “surface/underground work” remained unexplained. The factor lead the authors to introduce unusual cross-product terms of location and smoking in adjustment procedures and even caused the authors to hypothesize that high REC exposures are protective against lung cancer excess risks due to smoking. To understand the reliability of these conclusions, we should ask basic questions about the data collection process in DEMS: Did the mortality follow-up procedures suffer from errors like those that affected the NCI formaldehyde cohort study? Are the REC and/or smoking data reliable, and are these data collected/constructed in such a way that the procedures allow valid comparisons between surface and underground workers? Without clarifying the issues raised in this Commentary the Diesel Exhaust in Miners Study remains to be difficult to interpret.

## Background

Two risk analyses articles [1,2] about the US Diesel Exhaust in Miners Study (DEMS), were recently published in the Journal of the National Cancer Institute (JNCI). DEMS is an impressive epidemiological investigation organized by the National Institute for Occupational Safety and Health (NIOSH) and the National Cancer Institute (NCI). The authors have to be congratulated for this outstanding research project on the association between occupational diesel exhaust exposures, measured as long-term respirable elemental carbon (REC) estimates, and lung cancer mortality in a large cohort of US miners. The researchers summarized the main finding of DEMS as follows: “To our knowledge, this is the first report of a statistically significant exposure-response relationship for diesel exposure and lung cancer based on quantitative estimates of historical diesel exposure with adjustment for smoking and other potential confounders” [2]. Thus, these two publications [1,2] are expected to have considerable impact on the evaluation of the carcinogenic potential of diesel exhaust and, furthermore, on occupational and environmental limit value discussions related to diesel motor emissions and particle exposures.

Although this important study was well designed and performed, some issues may deserve a deeper scientific discussion. In the following I’d like to instigate such discussions while mainly focussing on methodological subtleties within DEMS. However, although subtleties, these methodological aspects may have substantial relevance for a reliable interpretation of the study and also for a justifiable application of study results in future evaluations of diesel exhaust. Because a Letter to The Editor and/or a Commentary on DEMS was not acceptable for the Editor of JNCI if the comments on the NCI formaldehyde cohort mortality study (cp. point 1 below) were not eliminated I decided to withdraw my submission from JNCI and to publish independently.

## Main text

In the following I will outline seven issues that deserve a deeper scientific discussion and should be addressed by the DEMS researchers.

1) The authors [1] reported an overall lung cancer SMR of 1.26. Internal analyses showed large increases of relative risks (RR) across estimates of REC exposure: Up to a 5fold or even 7fold lung cancer risk at high exposures (see abstracts of [1] and [2]). Such high RR estimates appear to be compatible with an overall SMR = 1.26 only, if there are deficits of lung cancer deaths among the low exposed (unfortunately, this cannot be justified directly because the authors did not report SMRs across REC exposure categories). If so this resembles the situation of formaldehyde epidemiology where an NCI cohort study [3] showed a pronounced increase of leukemia RRs in internal analyses while this increase was mainly based on a deficit of deaths among the low exposed [4]. Later it became clear that the mortality follow-up of the NCI study was relevantly incomplete [5,6]: NCI researchers had missed about 1000 deaths out of 9500. These deaths were missed proportionately more among the low exposed workers. This differential mortality follow-up error produced a substantial upward bias in risk estimates across exposure estimates [7]. Because the DEMS [1,2] was based on an evaluation of the US National Death Index Plus and the Social Security Administration death files like the NCI formaldehyde cohort study [3] and appears to have a similar structure of death deficits in the cancer endpoint of interest among the low exposed, the question arises whether the mortality follow-up of the Diesel Exhaust in Miners Study is really complete. Up to now NCI researchers neither explained why they failed in the mortality follow-up of the formaldehyde cohort and why this error was differential nor did they correct other obviously incomplete investigations [8] although this was urgently asked for [7]. Thus, the basis of US mortality studies applying follow-up procedures like those used in the NCI formaldehyde cohort study [3] remains dubious and this sheds doubt on the Diesel Exhaust in Miners Study also.

2) The DEMS [1,2] distinguished between “surface only work” and “ever underground work”. Table 2 in [1] reported on the lung cancer SMRs taking age, calendar time, gender, race and state into account. These mortality statistics differed unexpectionally between “surface only work” (SMR = 1.33) and “ever underground work” (SMR = 1.21). For the sake of clarity, I fitted a Poisson model to the data and it returned a ratio of 1.1 with a 0.95-CI of 0.83 - 1.46 and p-value of 0.51 [9]. However, the authors [1] reported a remarkably different finding after a further adjustment for REC exposure in the Cox models (all other covariates were identical to those in the SMR analyses): the ratio increased to 1.9 (range: 1.64-2.28, depending on the Cox model specification). It is perplexing that after adjustment for REC exposure estimates such a large risk factor between “surface only work” and “ever underground work” became apparent that went unnoticed without adjustment for REC exposure in the SMR analyses. However, even this remarkably high RR value of about 2 underestimates the strength of the risk factor “surface work vs. underground work” in a model adjusting for REC exposure because the indicator variable was not set up in an optimal way (see point 3). In the case–control study [2] this puzzling risk factor remained and could not be explained by a difference in smoking habits or any other covariate differences between surface and underground workers. It became even more confusing because a significant interaction between smoking effects and location of work was reported: Smoking was described to have a larger effect on lung cancer mortality when working on surface (i.e., a modification of the smoking effect by location and of the location effect by smoking was found). In all final models of the case–control study [2] REC exposure risk estimates were adjusted by such unusual cross-product variables of the two potential confounders whereas the baseline terms smoking and location were excluded from the model equations simultaneously. The coefficients of these models are difficult to understand. In particular, because the authors [2] missed to present modelling results as usually reported on in epidemiological studies: results after simultaneously adjusting for the potential confounders smoking and location – but without including interaction terms of these potential confounders. Such results were helpful for comparisons. Even more basic questions remained unanswered: what are the ORs for REC exposure after controlling for only those variables used in SMR calculations [2], and what are the ORs for smoking in underground and surface workers without adjustment for REC exposure?

3) The distinction between surface and underground work is obviously of major importance for an understanding of this study. The authors [1] clearly stated that those Cox models that followed their a-priori defined analysis plan did not show any convincing association between REC exposure estimates and lung cancer mortality. After subdividing the cohort into “surface only workers” vs. “ever underground workers” or after adjusting for a “surface only/ever underground work” indicator the Cox models returned pronounced dose–response relationships with REC exposure estimates. However, neither this subdivision nor the indicator variable made use of all information available. According to the description of the exposure assessment process a REC estimate was allocated to every person-year. And because jobs and REC exposures differed between surface and underground work a reliable exposure assessment should take account of workers’ location by year (which I assume was done, otherwise the exposure estimates were obviously inaccurate). Thus, the information whether a worker is on surface or underground should have been available for every person-year. Surprisingly, the authors [1,2] used this information only in part. They derived the variable “ever underground work” and defined it in a time-dependent manner. It had been more natural, easier to interpret and more complete to analyze the time-dependent variable “underground work” instead. Because the distinction between surface and underground work is central to this study the authors should have better used the full information available.

4) The REC exposure risk estimates differed with location [1]: the authors reported a twenty times higher excess risk per ug/m3-y on “surface only” in comparison to “ever underground”. After taking logs of exposure the “surface only” REC coefficient was about twice the one calculated for workers “ever underground”. The researchers [1] tested the differences of the effect estimates (significant on the log scale, not significant on the linear scale) but neither reported how they performed the tests nor did they show any details of the results. The usual way to perform such a test is to add an interaction term “REC exposure x location” to the models. It is surprising that the authors [1,2] did not present such interaction models whereas they introduced interaction terms among covariates in the case–control models [2]. Such interaction analyses on REC and location may inform us whether the REC effect estimates are homogeneous across location and, thus, whether the REC findings of the DEMS can be generalized.

5) The exposure assessment procedures were criticized to suffer from a considerable uncertainty [10]. The authors responded in detail while making clear that they do not believe and never claimed that the approach is without error. They agreed that imprecision exists in their exposure estimates [11]. Thus, the REC exposure intensity values allocated to each miner and person-year are no exact data describing the truth correctly but should be understood as exposure estimates with uncertainties. However, the DMES analyses [1,2] applied the exposure data as if fixed and without error. A more reliable analysis should cover these exposure uncertainties quantitatively by simulation procedures [12] or Bayesian analyses [13]. Uncertainties related to the covariate data, e.g. smoking habit information, can also be taken into account [14]. These extended and more realistic analyses may help to decide whether the unavoidable uncertainties of the exposure estimates and of the covariate data may have a relevant effect on the study findings or not.

6) In the following I list some minor points/questions, but they are worth mentioning:

Was the study censored at an age when death certificates are generally considered to be less reliable, e.g., at an age of 85 y? (e.g., [15])

Was the effect of potential confounders on the REC-OR evaluated on a single basis or simultaneously for all potential confounders of interests? (the authors referred to a change in estimate criterion < 10%, [2])

Was missing data always analyzed as a data category although this may have lead to distortions? [16]

How are the smoking habits distributed across facilities? (at the trona mines smoking was prohibited, [2])

How do the results change if those workers are dropped from analyses who were hired at an age greater than 40 years? (these miners had a higher probability of prior occupational exposures to carcinogens, [1])

How do the curvilinear relationships and goodness of fit statistics look like if more general analytical procedures are applied like fractional polynomials [17] or spline regression [18] and evaluated by information criteria [19]?

7) Both papers [1,2] cited and emphasized the German potash miner cohort study [20]. However, a new and different interpretation of this epidemiological investigation is indicated because a more detailed analysis has been performed taking prior exposures in Uranium mining into account [21]. In this updated analysis the OR per mg/m3-y of total carbon exposure was 1.02 (0.95-CI: 0.80-1.31), and high exposed workers showed an unexceptionable OR = 0.98 (0.95-CI: 0.61-1.58) in comparison to low exposed. Excess lung cancer mortalities were only indicated at very high cumulative exposures to diesel motor emissions. An international publication is currently under peer review.

## Discussion

In summary, the Diesel Exhaust in Miners Study [1,2] described remarkable exposure-response relationships between REC estimates and lung cancer mortality - conditional on a pronounced effect of surface vs. underground work on lung cancer risk. However, if this risk factor is ignored the estimated REC-lung cancer association is attenuated substantially. Unfortunately, the factor “surface/underground work” remained unexplained even in the case–control study [2]. It is worth noticing that the authors [1,2] relied on this risk factor in their main analyses. This lead them to introduce unusual cross-product terms of location and smoking in adjustment procedures [2] and even caused the authors to hypothesize that high diesel motor emissions are protective against lung cancer excess risks due to smoking and vice versa [2]. Although the authors listed mechanistic considerations in favour of this hypothesis such implications are surprising. Thus, to understand the reliability of these conclusions, we should go back and ask more basic questions about the data collection process: Did the mortality follow-up procedures suffer from errors like those that affected the NCI formaldehyde cohort study [3]? Are the REC and/or smoking data reliable, and are these data collected/constructed in such a way that the procedures allow valid comparisons between surface and underground workers and analyses across these groups?

## Conclusion

The researchers should be congratulated for the impressive DEMS investigation. However, without clarifying the major issues raised in this Commentary the Diesel Exhaust in Miners Study [1,2] remains to be difficult to interpret.

## Competing interests

The author is member of the research committee and scientific advisory group of EUGT (http://www.eugt.org/) and received research grants from EUGT for a project on the effectivity of low-emission zones.

## Authors’ contributions

PM drafted the manuscript. No other contributors were involved.
